# Three-Dimensional Bioprinting of Human Organs and Tissues: Bioethical and Medico-Legal Implications Examined through a Scoping Review

**DOI:** 10.3390/bioengineering10091052

**Published:** 2023-09-07

**Authors:** Giovanna Ricci, Filippo Gibelli, Ascanio Sirignano

**Affiliations:** Section of Legal Medicine, School of Law, University of Camerino, IT-62032 Macerata, Italy; giovanna.ricci@unicam.it (G.R.); ascanio.sirignano@unicam.it (A.S.)

**Keywords:** bioprinting, tissue engineering, precision medicine, bioethics

## Abstract

Three-dimensional bioprinting is a rapidly evolving technology that holds the promise of addressing the increasing demand for organs, tissues, and personalized medicine. By employing computer-aided design and manufacturing processes, 3D bioprinting allows for the precise deposition of living cells, biomaterials, and biochemicals to create functional human tissues and organs. The potential applications of this technology are vast, including drug testing and development, disease modeling, regenerative medicine, and ultimately, organ transplantation. However, as with any groundbreaking technology, 3D bioprinting presents several ethical, legal, and regulatory concerns that warrant careful consideration. As the technology progresses towards clinical applications, it is essential to address these challenges and establish appropriate frameworks to guide the responsible development of 3D bioprinting. This article, utilizing the Arksey and O’Malley scoping review model, is designed to scrutinize the bioethical implications, legal and regulatory challenges, and medico-legal issues that are intertwined with this rapidly evolving technology.

## 1. Introduction

Bioprinting, more specifically 3D bioprinting, is an innovative technology that has the potential to revolutionize the fields of tissue engineering and regenerative medicine. This technique involves the use of 3D printing technology to create live tissues and organs, which could be utilized for transplantation, drug testing, and disease modeling. The fundamental technical–biological basis of 3D bioprinting lies in the combination of computer-aided design (CAD) and biological materials to create three-dimensional structures. This process begins with the creation of a digital model or blueprint, which is typically based on medical imaging data such as MRI or CT scans. This model serves as a guide for the bioprinter, which deposits layers of biological material, often in the form of a bioink, to build up the desired structure. Bioinks typically consist of a mixture of cells and a supportive material known as a hydrogel, which provides a structure for the cells to grow and proliferate. In order to ensure the survival and functionality of the printed tissues and organs, it is crucial to incorporate a vascular network into the structures. This can be achieved through the use of multiple print heads, which can deposit different types of cells and materials simultaneously. For example, one print head could deposit cells that will form the tissue, while another could deposit a sacrificial material that will later be removed to form channels for blood vessels. Three-dimensional bioprinting technology is currently capable of producing relatively simple tissues such as skin, cartilage, and blood vessels. However, the creation of more complex organs such as the heart or liver remains a significant challenge due to the complexity of their structures and the need for multiple types of cells to be organized in a precise manner.

The biological origin of cells used for 3D bioprinting of organs and tissues is a topic of paramount importance in regenerative medicine and tissue engineering. Cells are the foundation of bioprinting, dictating the characteristics of the bioprinted tissue or organ. Frequently, differentiated cells or cell lines are employed; however, stem cells are expected to play a pivotal role in the advancement of this technology. There are three types of stem cells that can form the ink of 3D bioprinting:

1. Mesenchymal stem cells (MSCs): They include stem cells derived from bone marrow (BM-MSCs), umbilical cord (UC-MSCs), and adipose tissue (ADSCs).
2. Induced pluripotent stem cells (iPSCs): They are stem cells derived from skin or blood cells that have been reprogrammed back into an embryonic-like pluripotent state. This reprogramming allows iPSCs to develop into any type of human cell.
3. Human embryonic stem cells (hESCs): They were first derived in 1998 from the inner cell mass of blastocyst-stage embryos. These unique cells possess the ability to differentiate into all tissues in the human body, signifying their pluripotency; however, the initial process of deriving hESCs implies the destruction of human embryos involved.

There are also a number of technical challenges that need to be overcome in order to improve the resolution and speed of bioprinting, as well as to ensure the long-term survival and functionality of the printed tissues and organs. These include the development of more advanced bioinks that can support the growth and differentiation of a wider range of cell types, the optimization of the printing process to minimize damage to the cells, and the development of methods to mature and condition the printed tissues and organs before they can be used. 

Another fundamental technical issue is that of scaffolds, which are 3D structures that support the growth and proliferation of cells, acting as a temporary matrix for new tissue formation. These scaffolds can be made from a variety of biomaterials, including synthetic and natural polymers, ceramics, and composites [1]. Conventional scaffolds, often made from hydrogels, have been widely used due to their biocompatibility and ability to mimic the natural extracellular matrix. However, they often lack the mechanical strength and structural integrity required for tissue engineering applications. On the other hand, 3D printed hydrogel-based scaffolds, created using bioprinting techniques, offer several advantages. They provide precise control over the spatial distribution of cells and biomaterials, thereby enabling the fabrication of complex, heterogeneous structures that closely mimic native tissues. These scaffolds can also be designed to match the mechanical properties of the target tissue and have interconnected pore networks that promote tissue integration and regeneration. Moreover, 3D printed scaffolds offer the advantages of patient-specific customization and scalability, making them a promising direction for the future of tissue engineering and regenerative medicine [2,3,4]. Furthermore, when compared to other 3D scaffolding techniques, such as electrospinning, 3D printed scaffolds demonstrate superior performance for wound healing and tissue regeneration applications. Electrospun scaffolds often suffer from a lack of pore interconnectivity, which limits cell infiltration and tissue ingrowth. In contrast, 3D printed scaffolds can be designed with interconnected pore networks that facilitate cell migration, nutrient diffusion, and waste removal, promoting tissue integration and regeneration [5,6].

While 3D bioprinting represents a promising technology for tissue engineering and regenerative medicine, there is still a long way to go before it can be routinely used to produce functional organs for transplantation. In addition to the technical challenges (complexity and sophistication required to reproduce the intricate cellular architecture and functionality of human organs, maintaining the structural integrity of bioprinted organs, etc.), there are indeed significant commercialization issues. The first of these pertains to the scalability of the technology. While the concept of 3D bioprinting is impressive, scaling it up for mass production without compromising the quality and functionality of the printed tissues remains a formidable hurdle [7]. Additionally, the regulatory landscape for 3D bioprinted products is still evolving. The unique nature of these products, consisting of both biological and synthetic components, necessitates the development of new regulatory standards and frameworks [8]. Moreover, the cost of 3D bioprinting technology is high, which is a significant barrier to its widespread adoption. Various factors contribute to this, including the cost of bioinks, the complexity of the printing process, and the requirement for specialized equipment and personnel. However, with ongoing research and development, it is hoped that this technology will eventually provide a solution to the shortage of donor organs and transform the field of regenerative medicine.

Beyond the technical and commercialization challenges associated with effectively utilizing 3D bioprinting for organ and tissue creation, there lie even more complex bioethical and deontological issues. These multifaceted challenges will be the focus of our investigation, which we will attempt to explore in detail through an extensive scoping review.

The rationale for this scoping review is the identification of the prevalent bioethical and medico-legal challenges associated with the use of 3D bioprinting in medicine through a comprehensive literature review. Our aim was to decipher the various issues that complicate and hinder the application of this rapidly evolving technology in clinical practice. Given the broad and complex nature of these queries, we found the scoping review method to be the most suitable approach. A scoping review is a form of literature review that systematically maps the key concepts, types of evidence, and gaps in research related to a defined area or field by systematically searching, selecting, and synthesizing existing knowledge. It is particularly useful in complex or emerging fields where the diversity of literature prevents the feasibility of a more rigid systematic review. In conducting this review, we have employed the Arksey and O’Malley scoping review model, a universally recognized and suitable model for conducting high-quality scoping reviews. This model provides a methodological framework that allows for the identification, selection, and analysis of a broad range of research, including empirical studies and theoretical papers, thereby ensuring a comprehensive synthesis of the bioethical, legal, and regulatory challenges intertwined with 3D bioprinting technology.

## 2. Materials and Methods

In our scoping review, we employed the methodological framework proposed by Arksey and O’Malley [9] to systematically explore the bioethical and legal implications of 3D bioprinting of human tissues and organs. The process was conducted in five steps.

### 2.1. Step 1: Identifying the Research Question

The research question was identified using the Population/Concept/Context (PCC) framework, as recommended by the Joanna Briggs Institute [10]. Our research question was as follows: “What are the most relevant bioethical and legal implications of 3D bioprinting of human tissues and organs?” For the PCC framework, we defined the following terms:

- *Population*: Three-dimensional bioprinting users and stakeholders (e.g., researchers, healthcare professionals, patients, and regulators).
- *Concept*: Bioethical and legal implications of 3D bioprinting.
- *Context*: Human tissues and organ printing in medical research and clinical applications.

### 2.2. Step 2: Identifying Relevant Studies

A comprehensive literature search was conducted using the databases PubMed, Scopus, Web of Science, and Ovid Medline. We utilized a combination of keywords and MeSH terms, including “3D bioprinting”, “bioethics”, “legal implications”, “regulation”, “human tissues”, and “organ printing”. We decided to limit our research to articles written in English and published in the period from 2000 to today. We did not set any limits regarding the type of paper (including reviews, original research, book chapters, etc.). The grey literature was excluded from the research. The search strings were tailored for each database to ensure a comprehensive search. The search strings used in various databases were as follows:

- *PubMed*: (“3D bioprinting” [MeSH Terms] OR “3D bioprinting” [All Fields]) AND (“bioethics” [MeSH Terms] OR “bioethics” [All Fields] OR “legal implications” [All Fields] OR “regulation” [All Fields] OR “ethics” [All Fields]) AND (“human tissues” [MeSH Terms] OR “human tissues” [All Fields] OR “organ printing” [All Fields] OR “bioprinting” [All Fields]) AND (“2000/01/01” [PDAT]: “2023/12/31” [PDAT]) AND “English” [Language].
- *Scopus*: TITLE-ABS-KEY ((“3D bioprinting” OR “organ printing”) AND (“bioethics” OR “legal implications” OR “regulation” OR “ethics”)) AND PUBYEAR > 1999 AND PUBYEAR < 2024 AND LANGUAGE (“English”).
- *Web of Science*: TS = ((“3D bioprinting” OR “organ printing”) AND (“bioethics” OR “legal implications” OR “regulation” OR “ethics”)) AND PY = (2000–2023) AND LA = (English).
- *Ovid Medline*: (“Bioprinting.mp” OR “Organ printing.mp”) AND (“Bioethics.mp” OR “Legal implications.mp” OR “Regulation.mp” OR “Ethics.mp”) AND English language AND yr = “1999–2024”.

### 2.3. Step 3: Selecting Studies

**Figure 1:**
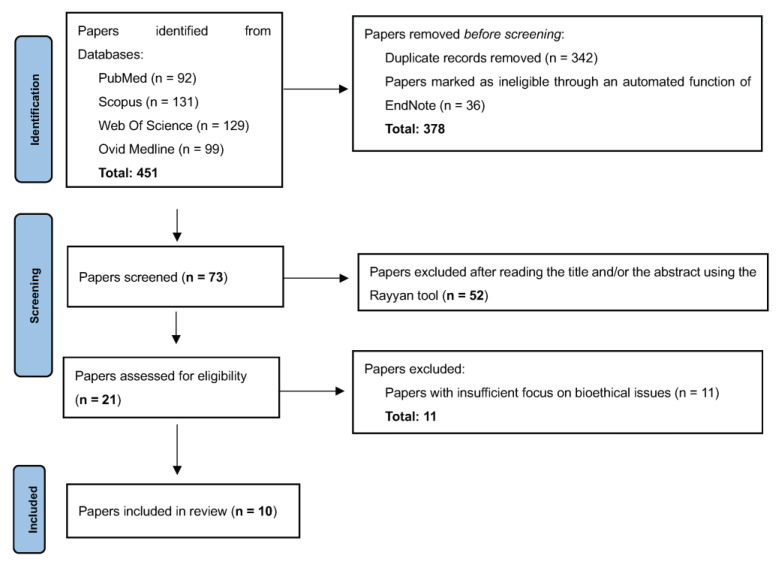
The selection process for articles included in the review.

To start, we used EndNote software to perform an initial review of the 451 articles pulled from the four databases. The software’s automated function helped us remove duplicates and articles that were clearly not relevant to the review’s objective, narrowing the selection down to 378 articles. These were then independently and anonymously scrutinized by the authors using the Rayyan tool (a web-based application designed to aid researchers in the process of systematic reviews, primarily for screening and selecting relevant literature); in this step, we decided whether to include or exclude articles based on their title and/or abstract. After the Rayyan screening, we further evaluated the 21 remaining articles by conducting an in-depth review of their full texts. Despite their focus on the bioethical implications of 3D bioprinting, 11 articles were excluded as they only touched on the topic tangentially and did not offer any substantial insights addressing the review question. Thus, only the remaining 10 articles were incorporated into the review. Figure 1 provides a visual representation of the various stages of the selection process.

### 2.4. Step 4: Charting the Data

In our pursuit to answer the review question, we employed a data mapping form in concert with Excel software to acquire the necessary data. We extracted the following details from the selected articles:

- Title of the article;
- Publication year;
- Geographical context (the country of the author’s or authors’ affiliation);
- Type of article;
- Major bioethical and medico-legal issues pertaining to 3D bioprinting.

The title of the paper provided a quick overview of the study’s primary focus. The year of publication and the origin of the author(s) were key in placing the article within its respective technological and socio-cultural context. The classification of the article played a vital role in understanding the scope of the research. The main findings enlightened us with the ultimate conclusions drawn in the article.

### 2.5. Step 5: Collating, Summarizing, and Reporting the Results

We report the results of the review in a schematic form in Table 1.

## 3. Discussion

As the literature review has shown, safety concerns are paramount. The complexity and unpredictability of the 3D organ printing process, including issues such as biomaterial degradation, tissue integration, biocompatibility, and continuous tissue synthesis during material degradation, pose significant challenges. The potential for irreversible risks, such as cancer and dislodgement and migrations of the implant, is a further complication that defies easy resolution [12,13,16,18,21,22]. The impossibility of testing the safety of the organ before implantation, due to the organ being custom-made for the person in whom it will be implanted and therefore unable to be tested on others, is a further obstacle. The extreme danger or impossibility of reversing organ implantation only adds to the dilemma. Adding to the potential risks of 3D bioprinting, the utilization of xenogeneic cells, which are derived from species different from humans, can lead to infectious and immunological risks. The introduction of these foreign cells could potentially trigger immune responses, resulting in organ rejection or graft-versus-host disease. Furthermore, there is the possibility of cross-species disease transmission, which can introduce new pathogens into the human population, posing a significant public health concern [23,24]. Furthermore, one of the core challenges in implementing 3D bioprinting technology lies in the integration of stem cells with scaffolds. Scaffolds, which serve as a three-dimensional framework for the cells, are crucial in tissue engineering as they provide support and guide the growth of new tissues. Various types of scaffold formulations are used, such as hydrogels, micro- and nanofibers, and micro- and nanospheres. Each type of scaffold formulation presents certain advantages and disadvantages. For instance, hydrogels facilitate cell survival and proliferation, while microfibers may offer a more desirable time course for drug delivery. These different scaffolds can also be combined to create novel hybrid materials, often leveraging the unique benefits of each formulation to optimize cell survival and drug delivery. However, the challenge lies in finding the right balance and integration for these diverse materials to ensure successful bioprinting and tissue growth [25].

As of now, international regulatory bodies like the Food and Drug Administration (FDA) and European Medicines Agency (EMA) have not established specific guidelines for 3D bioprinted organs and tissues. The FDA has been proactive in the realm of 3D printed medical devices, having cleared over 85 such devices for patient use, but they have not yet dealt with 3D printed organs as they are not a reality at this time [26]. Actually, in a 2017 document [27], the FDA expressed opposition regarding the possibility of using organs and tissues obtained through the process of 3D bioprinting, stating *“… Biological, cellular or tissue-based products manufactured using AM technology may necessitate additional regulatory and manufacturing process considerations and/or different regulatory pathways …”* and delegating the performance of the appropriate safety and efficacy checks to the Center for Biologics Evaluation and Research (CBER) [20]. Similarly, the EMA has not issued specific regulations for 3D bioprinted organs or tissues. Within the regulatory structure of the European Union, a product incorporating living cells or tissues is viewed as primarily exerting its effect through pharmacological, immunological, or metabolic actions. This perspective is held by a working group within the European Commission, which has classified bioprinted items as advanced therapy medicinal products, in accordance with Regulation (EC) 1394/2007 [20,21,22,23,24,25,26,27,28]. Both the FDA and EMA have an existing framework for Advanced Therapy Medicinal Products (ATMPs), which could potentially include 3D bioprinted organs in the future. Therefore, it is clear that while regulatory bodies are aware of the advancements in this field, the actual guidelines for 3D printed organs and tissues are yet to be defined. 

A second main ethical concern in 3D bioprinting is the origin of the biological materials used in the process. Currently, as explained in the introductory section, the sources of cells for bioprinting include adult stem cells (MSCs and IPSCs) and human embryonic stem cells (hESCs). The use of embryonic stem cells is particularly contentious, as it involves the destruction of human embryos, raising moral and ethical questions regarding the value and sanctity of human life. Conversely, the use of adult stem cells and induced pluripotent stem cells may be considered more ethically acceptable as this does not involve the destruction of embryos [11,12,13,14,15,16,17,18,19,20,21,22,23,24,25,26,27,28,29]. Some individuals view an embryo as a being with the same moral rights as an adult or a child, arguing from religious and moral standpoints that life starts at conception, making an embryo a person with rights and interests that need protection. They see the extraction of cells from a blastocyst to create an embryonic stem cell line as equivalent to committing murder. However, until proven otherwise, unless a blastula attaches to the uterine wall, it lacks the opportunity to evolve into a baby. Moreover, it is not unreasonable to argue that the embryo takes on a true “moral personality” at a stage of development following fertilization [30]. This is an enduring debate, the resolution of which appears scarcely within reach. Undeniably, it is crucial to maintain an open and ongoing discourse among science, society, and ethics to steer decisions in this intricate and perpetually changing field.

A third significant ethical issue that emerged from the review is affordability [13,14,15,16,17,18]. The cost of 3D bioprinters and consumables is high, often rendering this technology inaccessible to many and potentially exacerbating social inequalities [31]. The majority of affordable 3D bioprinters are constructed from modified fused deposition modeling 3D printer frames that are adapted for laying down biocompatible materials, with prices ranging from USD 13,000 to USD 300,000. Moreover, it is crucial to factor in the cost of supplies, which can vary between USD 3.85 and USD 100,000 per gram. This makes biomaterials costly and creates a barrier to accessibility for bioprinting, considering that high production costs translate into high costs for patients. In a bid to democratize this technology, a prototype of a cost-effective 3D bioprinter built from recycled materials and off-the-shelf electronics has been reported [32]. This approach, leveraging open-source methodologies and affordable materials, could make bioprinting more accessible, potentially bringing its benefits to low- and middle-income countries and bridging the economic divide in healthcare [3].

The matter of intellectual property (IP) is also fraught with difficulty [11,12,13,14,18]. Currently, 3D printing technology allows for the precise replication of complex structures, including human organs and tissues. These structures can be created using proprietary designs and methods, essentially transforming intangible knowledge into tangible, life-saving medical products. As such, the following question arises: who owns the intellectual property rights to these 3D printed organs and tissues? The conventional legal framework of intellectual property rights is proving to be inadequate for this emerging technology [33]. Traditional patent laws are designed to protect inventions that are novel, non-obvious, and useful. However, the specific case of 3D bioprinting blurs these clear lines. First, the concept of novelty becomes ambiguous when applied to biological structures that replicate the natural design of human organs. Second, the aspect of non-obviousness becomes difficult to define when the technology is merely replicating the existing biological reality. Third, while the utility of these printed organs and tissues is undeniable, it can be argued that the utility is derived not from the invention itself but from the natural function of the organ being replicated [34,35]. Another issue with the current patent system is the concept of “inventorship”. In the case of 3D bioprinting, multiple parties may be involved in the creation process, including the designer of the 3D printer, the scientist who develops the bioprinting method, the physician who implants the organ, and even the patient whose cells were used to create the organ. Determining who the true inventor is, in this case, can be a complex task, further exacerbating the challenges posed by bioprinting to the existing IP framework. Moreover, the current laws do not adequately address the moral and ethical concerns related to the ownership of 3D printed organs and tissues. For instance, if a company owns the patent to a certain organ design, does it also own the rights to the organ once it has been implanted in a patient? This question has profound implications for patient autonomy and bodily integrity. In the landmark case of Moore v. Regents of the University of California (1990) [36], it was held that a person does not retain ownership rights over cells once they have been excised from their body. Applying this principle to 3D bioprinting, it could be argued that a donor’s rights over their cells, and by extension the 3D bioprinted organ, are extinguished once the cells have been harvested. However, this raises ethical concerns, particularly where the donor’s cells are used for commercial purposes or in a manner contrary to the donor’s moral or religious beliefs. The legal frameworks governing ownership and control of tissues and organs vary globally. In the USA, the Uniform Anatomical Gift Act (2006) [37] permits individuals to donate their organs for transplantation, research, or education, but it does not expressly address the issue of 3D bioprinting. In contrast, in the UK, the Human Tissue Act (2004) [38] prohibits the use of organs for commercial purposes without explicit consent. These differing legal regimes reflect the wide range of ethical considerations that must be navigated in this emerging field. It is therefore imperative that a robust bioethical framework is developed to address these complex issues, balancing the need for scientific advancement with respect for individual autonomy and dignity.

That of informed consent is another significant issue [17,18]. As is well known, the provision of valid and informed consent is one of the prerequisites for the lawfulness of any medical practice. Firstly, the highly technical and specialized nature of 3D bioprinting poses challenges in effectively conveying the intricacies of the process to patients. Traditional medical or surgical procedures (think, for example, of the implantation of a body prosthesis) are comparatively much easier to explain, as they are grounded in tangible and familiar concepts. In contrast, 3D bioprinting involves advanced biotechnology techniques and concepts that may not be easily comprehensible to non-specialists, thus posing a barrier to effective informed consent. Secondly, informational activities often fall short in providing comprehensive disclosure about the biofabrication phase. While extensive details are generally provided about the production phase, where biological materials such as cells and tissues are harvested or cultivated, the biofabrication phase often lacks equivalent transparency. This phase, involving the actual creation of the artificial organ using 3D bioprinting, is pivotal in determining the success of the treatment. Nonetheless, it often remains shrouded in scientific jargon, potentially leaving patients uninformed about crucial aspects of their medical procedure. Lastly, as explained above, the nascent field of 3D bioprinting is characterized by a limited understanding of potential complications and outcomes. Owing to the highly individualized nature of 3D bioprinted implants, outcomes can be heterogeneous, thus limiting the ability to provide comprehensive and precise information to the patient. Consequently, this limited knowledge landscape compromises the comprehensiveness of informed consent, as patients cannot be fully informed about the potential risks and complications inherent in 3D bioprinting procedures.

Another aspect of bioethical relevance is the issue of clinical trials. It is difficult to think of conducting “traditional” clinical trials to test the effectiveness and safety of personalized materials obtained through 3D bioprinting. The main reason for this difficulty is that it would be ethically questionable to conduct tests on a population of subjects different from those for whom the material was customized, unless it is a matter of life-saving therapies. In other words, personalized materials obtained through 3D bioprinting are designed to uniquely fit the specific patient for whom they are intended. This means that testing these materials on a group of people different from those for whom they were created may not provide reliable results on the effectiveness and safety of these materials in real situations. Moreover, it would be ethically problematic to subject people to experimental therapies that have not been customized for them, unless they are therapies that could literally save their lives. There is also the fact that preclinical animal studies are unreliable models [19].

As 3D bioprinting technology advances, there is a risk of exploitation of vulnerable populations, particularly in the context of organ transplantation, and that is another issue [20]. The scarcity of organs for transplantation has led to the emergence of organ trafficking and transplant tourism, where individuals from wealthy countries acquire organs from poor and vulnerable individuals in developing nations. The commercialization of 3D bioprinting may exacerbate this problem by creating a market for biofabricated organs, potentially leading to further exploitation of impoverished populations as sources of biological materials.

Then there are the religious issues, concerning the moral and theological implications of manipulating life [18]. Within Christianity, opinions on bioprinting vary. Some Christian scholars argue that 3D bioprinting aligns with the concept of stewardship, where humans are called to responsibly care for the physical world. However, others believe that bioprinting encroaches upon divine creation and disrupts the natural order ordained by God. Scriptural analysis from the Bible, such as Genesis 2:7, is often invoked to support these diverse viewpoints [39]. Islamic perspectives on 3D bioprinting are multifaceted. Scholars emphasize the importance of preserving human life and advocate for the use of technology to alleviate suffering. However, concerns arise regarding the creation of organs or tissues that may challenge theological concepts such as the role of Allah as the sole creator. In the Jewish tradition, debates surrounding bioprinting primarily revolve around the sanctity of life and the concept of Pikuach Nefesh, which prioritizes saving lives. While some Jewish scholars argue that bioprinting advances this principle, others contend that it may undermine the idea of divine creation [40].

There is then another significant ethical issue, that of human identity [15]. The ability to create human organs and tissues through 3D bioprinting raises questions about the nature of human identity and the boundaries between humans and machines. As bioprinted organs become more sophisticated and closely resemble natural organs, it may become increasingly difficult to distinguish between what is considered “natural” and “artificial”. This blurring of boundaries may challenge our understanding of what it means to be human and raises ethical concerns about the potential consequences of these advancements on individual and societal levels.

The medico-legal issues should not be forgotten [17]. As with any medical intervention, the potential for complications and adverse outcomes associated with the use of bioprinted organs and tissues raises questions about liability. Determining who is responsible for any harm caused by bioprinted organs—whether it be the manufacturer, the surgeon, or another party—will be a complex and challenging issue. Establishing a clear legal framework for liability will be essential to ensure that those who suffer harm are adequately compensated and that medical professionals and manufacturers are held accountable for their actions.

## 4. Conclusions

In conclusion, the advent of 3D bioprinting technology represents a significant leap in the field of medical science, offering unprecedented opportunities for organ transplantation, regenerative medicine, drug testing and development, and disease modeling. However, the rapid growth and evolution of this technology have outpaced current regulatory, legal, and ethical frameworks, leading to a multitude of bioethical and legal implications that need careful scrutiny. Safety remains the paramount concern. As with any medical innovation, the potential risks and adverse outcomes associated with 3D bioprinted organs and tissues must be rigorously assessed and mitigated. In addition, there is a pressing need to establish comprehensive regulations to monitor the development and application of this technology, ensuring that it is used responsibly and ethically. The use of embryonic stem cells in 3D bioprinting raises fundamental ethical questions regarding the legitimacy of their use. It is crucial to facilitate informed, inclusive, and open dialogues that respect diverse perspectives and values while aiming for a consensus on this contentious issue. Access to care, an essential principle in healthcare ethics, is challenged by the possibility of 3D bioprinting becoming a luxury available only to the privileged few. Policymakers and stakeholders must strive to ensure equity of access and affordability, preventing the exacerbation of existing health disparities. Intellectual property issues arise with the potential for commercial exploitation of 3D bioprinted organs and tissues. Determining ownership and obtaining informed consent are complex issues that require legal clarity. The question of liability in the event of harm or damage also demands careful consideration. Religious and cultural concerns pertaining to 3D bioprinting need to be acknowledged and respected, ensuring that the technology aligns with societal values and norms.

As we navigate the uncharted territories of 3D bioprinting, it is imperative to establish robust, flexible, and inclusive ethical and regulatory frameworks that adapt to the evolving technological landscape. Such frameworks should promote the responsible development and use of 3D bioprinting while addressing the myriad of ethical, legal, and regulatory challenges it presents. This scoping review underscores the need for ongoing, interdisciplinary research and dialogue to inform these necessary frameworks, fostering an environment where technological progress complements, rather than conflicts with, our ethical and legal obligations.

## Figures and Tables

**Table 1:** Summary of articles included in the review.

Reference	Title	Year	Geographical Context	Article Type	Main Bioethical and Medico-Legal Issues Related to 3D Bioprinting of Human Organs
Vijayavenkataraman et al. [11]	3D bioprinting—an ethical, legal and social aspects (ELSA) framework	2016	Singapore	Review article	Questionable ethical legitimacy of destroying an embryo to produce embryonic stem cells Necessity and controversy of preclinical testing on animals for bioprinted organs and tissues Patient selection, informed consent, and transparency of funding in clinical trials Patentability of bioprinting and ownership of bioprinted products Difficulties related to the classification of bioprinted products for regulatory approval Society’s perception of organ bioprinting and the subject of bioprinted organs
Neely [12]	The Risks of Revolution: Ethical Dilemmas in 3D Printing from a US Perspective	2016	USA	Perspective article	Safety concerns Intellectual property concerns
Vermeulen et al. [13]	3D bioprint me: a socioethical view of bioprinting human organs a socioethical view of bioprinting human organs and tissues	2017	UK	Review article	Risk of social stratification (and thus inequality) Complexity and unpredictability of 3D organ printing process (issues such as biomaterial degradation and tissue integration, biocompatibility, and continuous tissue synthesis during material degradation) Potentially irreversible risks (cancer, dislodgement, and migrations of implant) Ownership and patenting of bioprinted organs
Ravnic et al. [14]	Transplantation of bioprinted tissues and organs: Technical and clinical challenges and future perspectives	2017	USA	Review article	Confidentiality issues (related to data acquired during the “digital scan” of the patient) Concerns about possession and control of the cell lines Difficulties in balancing the benefits for the many different stakeholders (patients, doctors, universities, and technology companies)
Patuzzo et al. [15]	3D Bioprinting Technology: Scientific Aspects and Ethical Issues	2017	Italy	Review article	Safety issues Questionable ethical legality of using biotechnology to manipulate human nature Criticism in providing information to the general population that fully explains the benefits and risks of the 3D bioprinting technique Ethical legitimacy of embryonic stem cell production through the destruction of embryos Alteration of human personality to the point of “dehumanizing” human individuals when using xenogeneic cells Problems of equity of access to care related to the high cost of technology
Gilbert et al. [16]	Print Me an Organ? Ethical and Regulatory Issues Emerging from 3D Bioprinting in Medicine	2018	Australia	Review article	Existence of potential and uncertain risk of harms Impossibility of testing the safety of the organ before implantation (since the organ is custom-made for the person in whom it will be implanted and therefore cannot be tested on others) Impossibility or extreme danger of reversing organ implantation Uncertainty as to how organ bioprinting will be effectively evaluated and approved for clinical use given the absence of specific regulations
Kirillova et al. [17]	Bioethical and Legal Issues in 3D Bioprinting	2020	Russia	Perspective article	Lack of a sufficiently adequate and comprehensive legal framework (regulating, e.g., liability profiles) Problems of equity with regard to accessibility of treatment limited to the wealthiest groups Concerns regarding the fact that informed consent pertains to the production of biological material rather than the biofabrication of the organ itself Doubts about the risk–benefit ratio with regard to 3D printing of ovaries (bioprinted ovary project) Again with reference to 3D printing of ovaries, the woman may feel an emotional obligation to receive the organ once printed even though she does not want it (since several years may pass between the time the ovary is removed for oncological reasons and the time the new 3D printed ovary can be implanted, and the woman may change her mind)
Datta et al. [18]	Ethical challenges with 3D bioprinted tissues and organs	2022	India–USA	Perspective article	Safety concerns Unpredictability of risks and consequent difficulties in performing an accurate risk–benefit analysis Concerns about the feasibility of providing a truly comprehensive information activity (given the presence of unknown risks) Ownership issues (can a donor claim ownership of his or her donated cells?) Concerns in terms of equity and accessibility Religious issues (organs that should not be 3D printed and cells from certain animals that should not be used)
Harris et al. [19]	Ethical and regulatory issues of stem cell-derived 3-dimensional organoid and tissue therapy for personalised regenerative medicine	2022	Australia	Perspective article	Safety issues (possibilities of harm from tissue necrosis or cysts; presence of differences between the structure or function of cultured and biological tissues and consequently possible problems of interaction with surrounding tissues) Inadequacy of preclinical animal studies for 3D bioprinting Difficulties in defining the “moral status” of 3D printed organs and tissues (simple body parts or something more?)
Rizzo et al. [20]	3D printing and 3D bioprinting technology in medicine: ethical and legal issues	2023	Italy	Review article	Safety issues (risk of developing cancer and zoonosis when using xenogeneic cells) Lack of homogeneity in international legislation Lack of international standards for the selection of medical materials for 3D bioprinting Risk of misuse of technology (bioterrorism or organ trafficking) Difficulties in conducting traditional clinical trials

## Data Availability

No new data were created or analyzed in this study. Data sharing is not applicable to this article.
